# Hyperammonemic Encephalopathy after Bariatric Surgery: A Two-Hit Vulnerability in Liver Disease

**DOI:** 10.14309/ctg.0000000000000892

**Published:** 2025-08-12

**Authors:** Mohammed Abdulrasak, Mostafa Mohrag, Ali M. Someili

**Affiliations:** 1 Department of Clinical Sciences, Lund University, Malmo, Sweden;; 2 Department of Gastroenterology and Nutrition, Skane University Hospital, Malmo, Sweden;; 3 Department of Medicine, Faculty of Medicine, Jazan University, Jazan, Saudi Arabia.

We read with great interest the article by Tujios et al on the association between prior bariatric surgery and increased severity of acetaminophen-induced acute liver failure (ALF). The observation that patients with prior bariatric surgery developed more severe encephalopathy (grade 3–4), despite lower acetaminophen ingestion and comparable biochemical liver injury, raises important questions about neurotoxicity susceptibility in this population (1).

We propose a potential explanatory framework grounded in a “two-hit” model. Roux-en-Y gastric bypass and other bariatric procedures, while metabolically beneficial, are increasingly recognized to confer distinct biochemical vulnerabilities. One such complication is noncirrhotic hyperammonemic encephalopathy, a well-documented but underappreciated entity in postbariatric patients (2).

Mechanistically, noncirrhotic hyperammonemic encephalopathy may arise due to small intestinal bacterial overgrowth, micronutrient deficiencies (particularly zinc), and sarcopenia, all contributing to impaired ammonia detoxification (2). Additionally, partial or latent urea cycle disorders—particularly ornithine transcarbamylase deficiency in heterozygous females—may be unmasked in the postbariatric setting (3). The result may be chronic, subclinical hyperammonemia in otherwise compensated individuals.

We hypothesize that such baseline ammonia accumulation constitutes a metabolic “first hit,” priming the central nervous system for exaggerated encephalopathy upon exposure to a “second hit,” such as liver injury from acetaminophen or chronic liver disease. Preliminary data in cirrhosis cohorts appear to support this: Trujillo et al reported increased rates of hepatic encephalopathy among cirrhotics with prior bariatric surgery, even in the absence of advanced liver dysfunction (4).

In light of these findings, we suggest clinicians maintain a low threshold for ammonia monitoring in postbariatric patients with altered mental status or hepatic decompensation. Ammonia-lowering therapies—such as lactulose, rifaximin, and micronutrient repletion (e.g., zinc or L-carnitine)—may be warranted even in early or mild liver disease, though current evidence is extrapolated primarily from cirrhotic populations (2,5).

In sum, we believe that prior bariatric surgery may confer a latent risk for neurotoxicity through mechanisms distinct from classical hepatic insufficiency. The proposed two-hit hypothesis warrants further prospective study to clarify risk stratification, screening strategies, and treatment thresholds in this growing and vulnerable patient population.

## CONFLICTS OF INTEREST

**Financial support:** None to report.

**Potential competing interests:** None to report.
